# Poor Health Conditions among Brazilian Healthcare Workers: The Study Design and Baseline Characteristics of the HEROES Cohort

**DOI:** 10.3390/healthcare10102096

**Published:** 2022-10-20

**Authors:** Tatiana de Oliveira Sato, Beatriz Suelen Ferreira de Faria, Bianca Biason Albuquerque, Fabio Leandro da Silva, Luiza Salvador Rohwedder, Renata Trivelato de Azevedo, Josiane Sotrate Gonçalves, Ludmilla Maria Souza Mattos de Araújo Vieira, Maria Isabel Triches, Rosângela Aparecida de Sousa, Viviane de Freitas Cardoso, Vivian Aline Mininel

**Affiliations:** 1 Physical Therapy Department, Universidade Federal de São Carlos, São Carlos 13565-905, Brazil; 2 Nursing Department, Universidade Federal de São Carlos, São Carlos 13565-905, Brazil

**Keywords:** cumulative trauma disorder, ergonomics, health occupations, nurses

## Abstract

This study was conducted to describe the health conditions (the psychosocial aspects, sleep quality, and musculoskeletal symptoms) among Brazilian healthcare workers in the context of the pandemic. Workers answered an online questionnaire, including the short version of the Copenhagen Psychosocial Questionnaire (COPSOQ II), the Pittsburgh Sleep Quality Index (PSQI), the Nordic Musculoskeletal Questionnaire (NMQ), and the Beck Depression Inventory (BDI). The most unfavourable psychosocial factors were work pace (61%; 95% CI: 52–69%), emotional work demands (75%; 95% CI: 67–82%), predictability (47%; 95% CI: 39–56%), work-family conflict (55%; 95% CI: 46–64%), burnout (86%; 95% CI: 78–91%), and stress (81%; 95% CI: 73–87%). Most workers (74%; 95% CI: 66–81%) were classified as poor sleepers. Musculoskeletal symptoms were frequent in the neck (64%; 95% CI: 55–72%), shoulders (62%; 95% CI: 54–70%), upper back (58%; 95% CI: 50–67%), and lower back (61%; 95% CI: 52–69%). Depressive symptoms were also highly prevalent (mild: 22%; 95% CI: 15–30%, moderate: 16%; 95% CI: 11–23%, severe: 8%; 95% CI: 4–14%). Most healthcare workers experience unfavourable psychosocial factors, poor sleep quality, as well as musculoskeletal and depressive symptoms. These findings underscore the urgent need to acknowledge and address psychological and physical distress to improve the personal and professional well-being of this population.

## 1. Introduction

Healthcare providers deal with physical and psychosocial demands at work due to long working hours, aspects related to patient management, a high level of attention, and shift work. Shift workers may also experience sleep imbalances [1,2]. Such aspects mean that healthcare workers are commonly affected by health problems, such as musculoskeletal symptoms and mental disorders, which can compromise their quality of life as well as the quality of the service provided [2].

Musculoskeletal symptoms are common in workers and have been associated with low control and high demand at work [3]. Moreover, mental disorders have received considerable attention due to the growing number of sick-listed workers [4]. Indeed, depression is expected to become one of the leading causes of illness in the world by 2030.

Depressive symptoms are more prevalent among women, who also constitute the majority of healthcare workers [5,6,7]. Factors associated with depression include back symptoms [8], BMI ≥ 25 kg/m^2^ [9], poor sleep or insomnia [7,10], high work demand and low control [11], excessive workload (>60 h) [12,13], job insecurity [12], stress [11], low income [14], shift work [13], burnout [14,15], low social support [16], physical violence [13], and a lack of physical activity [13]. Healthcare workers may be exposed to all these factors in addition to the emotional burden derived from the suffering and pain of patients and family members [14]. Despite being the main actors responsible for sustaining the Brazilian public healthcare system and playing an essential role in assisting the population, healthcare workers are subjected to several factors that can exert a negative impact on their health and, consequently, compromise their performance and the quality of care.

Repercussions of this context reflect on workers’ loss of quality of life, self-responsibility for seeking treatment, increasing family costs, frustration, and suffering and on employer’s costs due to absenteeism and work limitations on workers’ return-to-work, as well as the poor delivery of care and patients’ dissatisfaction. Additional repercussions are apparent for co-workers’ practice owing to increased workloads and job demands, and on all of society due to the associated compensation system and public health costs. 

The context of the COVID-19 pandemic brought to light the harsh reality of healthcare workers, who suffer (and die) under precarious working conditions, professional devaluation, a lack of institutional support, irregular schedules, and strained employment relationships [17]. Therefore, the aim of the present study was to describe the health conditions of Brazilian healthcare workers using the baseline measurements of the HEROES cohort, with an evaluation of the population’s psychosocial aspects, sleep quality, musculoskeletal symptoms, and depressive symptoms.

## 2. Materials and Methods

### 2.1. Study Design

We conducted a cohort study with a prospective 12-month follow-up. The study design and baseline characteristics of the sample are presented in this article and followed the checklist from the Strengthening the Reporting of Observational Studies in Epidemiology (STROBE) statement [18] and the Checklist for Reporting Results of Internet E-Surveys (CHERRIES) [19].

### 2.2. Recruitment Process

The recruitment process for the target population included advertisements in the local press, social media (Facebook, Instagram, LinkedIn, WhatsApp, and Youtube), and institutional emails available on the websites of healthcare organisations. We conducted convenience sampling based on the voluntary responses of the participants.

The inclusion criteria included Brazilian workers 18 years of age or older who performed healthcare activities at any service offered by the public healthcare system during the pandemic of COVID-19. Students, interns, retirees, and inconsistent or repeated data were excluded from the sample.

### 2.3. Ethical Aspects

This study was approved by the Brazilian Research Ethics Committee (certificate number: 39705320.9.0000.5504), and all participants provided informed consent. The research development respected the current ethical standards and resolutions.

### 2.4. Participants

**Figure 1:**
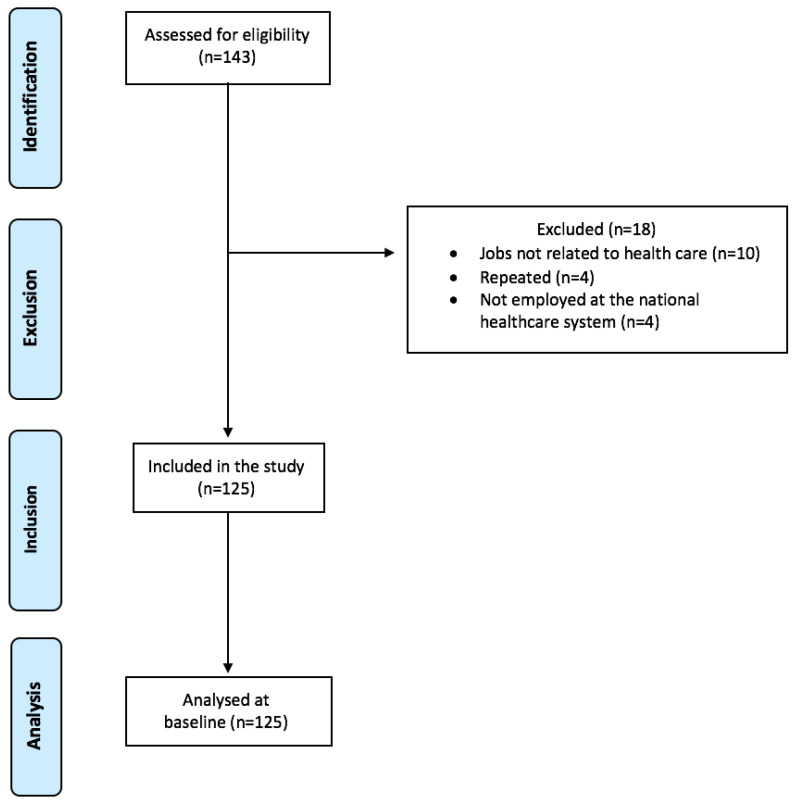
Flowchart of the participant selection process in accordance with Strengthening the Reporting of Observational Studies in Epidemiology (STROBE).

One hundred and forty-three workers answered the questionnaires at baseline, but only 125 met the eligibility criteria and were included in the HEROES cohort. The reasons for exclusion were: not working with healthcare activities at the time (n = 10), duplicate answers (n = 4), and not being employed by the public healthcare system (n = 4) (Figure 1).

### 2.5. Data Collection

#### 2.5.1. Baseline Measurements

Five questionnaires addressing the outcomes of interest were employed. These included: sociodemographic and occupational characteristics, with questions about sex, age, marital status, education, employment status, occupation, healthcare sector of work and lifestyle; the short version of the Copenhagen Psychosocial Questionnaire (COPSOQ II-Br); the Pittsburgh Sleep Quality Index (PSQI); the Nordic Musculoskeletal Questionnaire (NMQ); and the Beck Depression Inventory (BDI).

The psychosocial aspects at the workplace were evaluated through the short version of the COPSOQ II translated to Brazilian Portuguese (COPSOQ II-Br) [20]. It has 40 items addressing quantitative work demands, work pace, emotional work demands, influence on work, new skill development, meaningful work, commitment to the workplace, predictability, appreciation and recognition, role clarity, leadership quality, social support from superiors, job satisfaction, work-family conflict, management or worker trust, justice and respect, self-rated health, burnout, stress, unwanted sexual attention, threats of violence, physical violence, and bullying. All items were evaluated on a Likert scale, from 0 to 3 or 4, except for the offensive behaviour domain, which had yes or no questions. The final score was determined by the sum of the items in each of the domains. For each dimension, the score classifies the work environment as “safe”, “requires attention”, or “poses risks” [21].

The Pittsburgh Sleep Quality Index (PSQI) developed by Buysse et al. [22] was administered to discriminate between the “good sleepers” and “poor sleepers”. This index has been translated and adapted to Brazilian Portuguese [23] and consists of 19 self-administered questions. The questions are grouped into seven components with weights distributed on a scale from zero to three: (i) subjective sleep quality, (ii) sleep latency, (iii) sleep duration, (iv) habitual sleep efficiency, (v) sleep disorders, (vi) use of sleeping medication, and (vii) daytime dysfunction. The scores are summed to produce the total, which ranges from 0 to 21, with higher scores denoting poorer sleep quality. A total score higher than five points indicates that the individual has difficulties regarding at least two components or moderate difficulties regarding more than three components.

The Nordic Musculoskeletal Symptom Questionnaire (NMQ) is a validated tool designed to investigate musculoskeletal symptoms in nine body regions [24]. The questionnaire assesses symptoms in the previous 12 months and the previous 7 days, the occurrence of functional disability, and the search for assistance from a healthcare provider in the previous 12 months. The answers are dichotomous. This questionnaire is widely used in the field of occupational therapy and contributes to the identification of workers with pain. The Brazilian version adapted by Barros and Alexandre [25] was used for the present investigation.

The Beck Depression Inventory (BDI) is a self-administered scale used to assess symptoms of depression. The BDI is composed of 21 items addressing symptoms and attitudes to assess depression in clinical and non-clinical patients. Each item is scored on a four-point scale ranging from zero (no symptoms) to three (severe symptoms). The respondents rate the items based on their condition in the previous two weeks as well as the day on which the test was administered. If multiple statements describe a condition, the participant is asked to choose the answer with the highest number on the scale. The total score is calculated by summing the scores of the 21 items and ranges from 0 to 63. Although there are no arbitrary cut-off points for the diagnosis of each category of depression, the following ranges of scores indicate a specific category: 0 to 13 points—an absence of depression, 14 to 19 points—mild depression, 20 to 28 points—moderate depression, and 29 to 63 points—severe depression [26].

#### 2.5.2. Follow-Up

**Figure 2:**
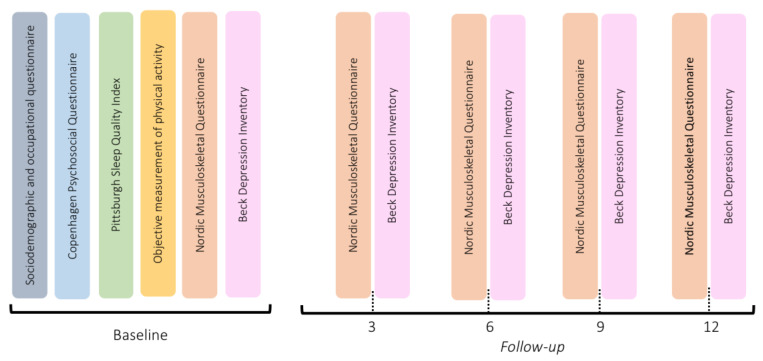
Questionnaires applied to each stage of the study.

Five data points were collected over a 12-month period, considering the baseline and four further measures (at 3, 6, 9, and 12 months), using the questionnaires described in Figure 2.

### 2.6. Procedures

All questionnaires were included in Google Forms for data gathering without adaptations. The final version of the form consisted of 10 pages, and a progress bar was included for participants to keep track of their answers. We performed previous tests on this platform to determine the time required to answer all questionnaires and correct typographical errors. After this stage, we publicised the link through the recruitment strategies.

The form was open to anyone interested in responding. There was no incentive or remuneration to participate in the study. The baseline data collection started on 19 June 2021, and ended on 4 April 2022. The follow-up data collection started on 19 September 2021, and continues. Thus, the follow-up started before the baseline data collection was completed. All answers were electronically registered.

The inclusion and exclusion criteria were applied after the answers were collected. There were no incomplete questionnaires. No cookies or IP collections were used. The statement of informed consent was inserted into the forms, and a copy signed by the project supervisor was available for download.

### 2.7. Data Analysis

The data were organised into spreadsheets, and the personal information was replaced with an identification number which ascended according to the order of the responses. The time taken to answer the questionnaires was not measured since it was not relevant to the study.

The variables from the five questionnaires were analysed in SPSS software (version 26.0) through descriptive statistics (the absolute (n) and relative (%) frequencies, mean, and standard deviation (SD)). Bivariate associations were tested using the Chi-square test to verify the association between sociodemographic variables and the outcomes. The significance level was set to 5%.

## 3. Results

Most participants were female, between 31 and 40 years, white, married, without children, had a university degree, were of normal weight, were physically active, and had no diagnosed diseases or drug prescriptions. Most participants were on the nursing team (nurses, nursing assistants, or technicians), had more than five years of job seniority, worked at hospitals, worked 40 h per week, did not have more than one job, and received between three and six times the monthly minimum wage (Table 1).

**Figure 3:**
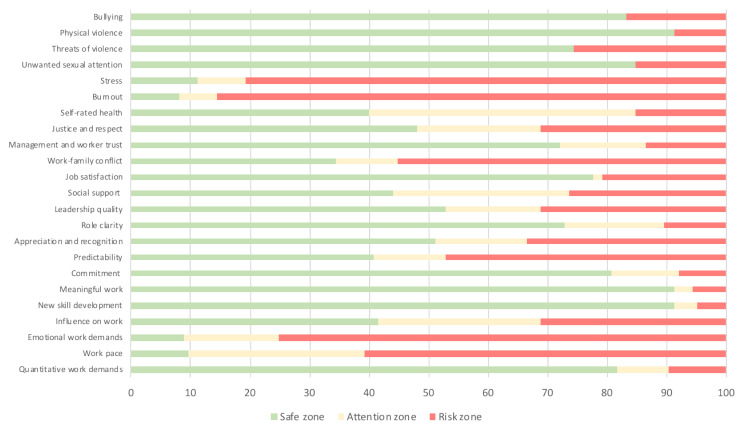
Percentage of psychosocial risks according to the dimensions of the COPSOQ II-Br (n = 125). Data are expressed as %.

The most favourable aspects were related to quantitative work demands, influence on work, new skill development, meaningful work, commitment to the workplace, appreciation and recognition, role clarity, leadership quality, social support from superiors, job satisfaction, management or worker trust, justice and respect, and offensive behaviours (unwanted sexual attention, threats of violence, physical violence, and bullying). The riskiest factors were work pace, emotional work demands, predictability, work-family conflict, burnout, and stress (Figure 3).

Thirty-two participants (25.6%; CI 95%: 19–34%) were considered good sleepers, and 93 (74.4%; CI 95%: 66–81%) were considered poor sleepers. Musculoskeletal symptoms were more frequent in the neck, shoulders, upper back, and lower back in the previous 12 months and the previous seven days. The lower back was the most affected region, and the neck was the region that caused most of the visits to healthcare providers in the HEROES cohort (Table 2). Depressive symptoms were also highly prevalent (mild: 22%, 95% CI: 15–30%; moderate: 16%, 95% CI: 11–23%; severe: 8%, 95% CI: 4–14%).

Significant associations were found between age and work pace (*p* = 0.02; higher risk: 31–40 years), unwanted sexual attention (*p* = 0.01; higher risk: 18–30 years), and symptoms on the lower back in the last 7 days (*p* = 0.03; higher risk: 31–40 years). Occupational groups were significantly associated with influence at work (*p* < 0.01; higher risk: nurse assistant/technician), neck, lower back, and wrist/hand disability (*p* < 0.01; higher risk: nurse assistant/technician), and symptoms on the shoulders in the last 7 days (*p* = 0.04; higher risk: dentist). Job seniority was significantly associated with recognition (*p* = 0.04; higher risk: 2–5 years), trust (*p* < 0.01; higher risk: more than 5 years), work-family conflict (*p* < 0.01; higher risk: 2–5 years), unwanted sexual attention (*p* < 0.01; higher risk: less than 2 years), shoulder disability (*p* = 0.02; higher risk: 2–5 years), lower back healthcare assistance (*p* = 0.03; higher risk: more than 5 years), symptoms on the foot in the last 7 days (*p* = 0.04; higher risk: less than 2 years), and depression symptoms (*p* = 0.02; higher risk: 2–5 years). Income was significantly associated with influence, predictability, recognition, trust, justice, role clarity, social support, satisfaction, threats of violence, bullying, and elbow, lower back, and knee disability (*p* < 0,05; higher risk: 1–3 MW). Educational level was significantly associated with influence (*p* < 0.01; higher risk: non-university), skill development (*p* < 0.01; higher risk: non-university), burnout (*p* < 0.03; higher risk: university), lower back, hand, and foot disability, and lower back healthcare assistance (*p* < 0.05; higher risk: non-university).

## 4. Discussion

The findings of the present study showed that Brazilian healthcare workers were subjected to several unfavourable psychosocial factors during the COVID-19 pandemic, such as an excessive work pace, high emotional work demands, low predictability, work-family conflict, burnout, and stress. Bivariate associations highlighted the most vulnerable groups, which are younger workers (unwanted sexual attention), nurse assistants or technicians (psychosocial risks and symptoms), dentists (shoulder symptoms), groups with the lowest income (psychosocial risks and symptoms), and groups with lower (psychosocial risks) and higher (burnout) educational levels.

Comparing these results to the findings from other studies, we found higher proportions of stress (81%) and burnout (86%) in our sample. Nine meta-analysis studies investigated the pooled prevalence of stress in healthcare workers and found rates ranging from 17% to 57% [27]. Burnout was evaluated in five meta-analyses, and the pooled prevalence ranged from 25% to 37% [28,29].

Offensive behaviours in the form of unwanted sexual attention, threats of violence, physical violence, and bullying affected 15%, 26%, 9%, and 17% of the healthcare workers in our sample, respectively. Such behaviours are quite frequent in Brazil and are mainly directed at female nurses. Pai et al. [30] and Vasconcellos et al. [31] showed that 3% and 6% of their samples reported the occurrence of unwanted sexual attention in the workplace. Threats of violence constituted the most common type of work-related violence and were reported by 43% [32], 49% [30], and 65% [31] of Brazilian healthcare workers in previous studies. The frequency of physical violence was also high in the present sample and similar to rates reported in other Brazilian studies, such as 3% [31] and 15% [30].

Thirty-two participants (25.6%) were considered good sleepers, and 93 (74.4%) were poor sleepers. Cotrin et al. [33] also found that 66% of Brazilian nurses reported sleep difficulties during the pandemic. Huang and Zhao [34] found that 24% of Chinese healthcare workers reported poor sleep quality. In a meta-analysis study of sleep disturbances during the COVID-19 pandemic, Jahrami et al. [35] found that 42% of healthcare workers faced this problem. In contrast, higher frequencies were reported in other studies that used the PQSI, as Proserpio et al. [36] found that 80% of healthcare workers reported poor sleep, and Stewart et al. [37] found that the prevalence of poor sleep was 96%.

These unfavourable psychosocial and sleep conditions may also be reflected in the high rates of musculoskeletal symptoms in the neck, shoulders, upper back, and lower back in the present sample. Moreira et al. [38] evaluated a similar sample of Brazilian healthcare workers and found a lower percentage of complaints for all body parts. In the context of the pandemic, El Far et al. [39] found that the prevalence of low back pain among healthcare workers was 81%, and the symptoms were associated with prolonged standing, awkward postures, shift work, overtime work, work pace, and insufficient rest. Arca et al. [40] found a similarly high frequency of symptoms in the previous 12 months using the NMQ (neck: 73%; shoulders: 56%; upper back: 68%; lower back: 71%).

The present findings indicate a higher rate of depressive symptoms in our sample (46%) compared to meta-analysis studies, in which the pooled prevalence in healthcare workers ranged from 12% to 37% [27,41]. However, a study conducted with Brazilian healthcare workers found a similar rate of depression, indicating that this problem could be more frequent in Brazil [42].

Such results could be related to the overwhelming impact of the pandemic in Brazil in terms of the number of cases, hospitalisations, and deaths. Most healthcare workers were on the frontline in the fight against the pandemic, suffering from stress due to difficult decisions that needed to be made, frequent contact with cases of suffering and death, the enormous pressure placed on the healthcare system, the need to provide care for many patients simultaneously, as well as the risk of becoming infected and contaminating relatives and close friends [17]. Brazil was the leading country in the number of cases of contamination and deaths among healthcare workers [43]. Moreover, infection with the SARS-CoV-2 virus presented a pattern of inequality, exerting a greater impact on healthcare workers with a low income and schooling [44].

Busch et al. [28] highlighted the need to acknowledge and address this psychological distress through communication and psychological support in order to reduce uncertainty, strengthen coping skills, restore a sense of control, enhance self-efficacy, and work through traumatic memories. Attention to these issues could help improve personal and professional well-being among healthcare workers during the context of the pandemic.

A hidden effect of the pandemic in Brazil was the deterioration of the health conditions of healthcare workers. These effects need to be investigated in the long term. Although the term “HEROES” was chosen to honour this cohort of Brazilian healthcare workers, our collective gratitude for the work carried out by these workers should also be manifested in actions of social valorisation and adequate working conditions.

The small sample size and the purposive sampling are important limitations of this study. Moreover, the online design may over-represent some categories of healthcare workers, as suggested by the greater participation of young workers with a university degree. One reason for the low participation rate could be the number of questions on the online form and the high workload of the participants.

## 5. Conclusions

Most healthcare workers experience unfavourable psychosocial factors, poor sleep quality, musculoskeletal symptoms, and depressive symptoms. These findings highlight the need to address psychological and physical distress to improve the personal and professional well-being of this Brazilian working population. Considering that workers’ health and safety is a systemic problem in organisations, there is an urgent need to take responsibility to promote better workplace conditions, fair work organisation, and horizontal decision-making, to reduce the imbalance of power, and to empower workers to create changes in their daily work processes. It is necessary to go beyond occupational risk management and think about how to foster happiness and well-being at work together with workers, employers, researchers, and society.

## Figures and Tables

**Table 1:** Personal and occupational characteristics of participants in the HEROES cohort (n = 125).

Characteristics	n	%
Sex		
Female	104	83.2
Male	21	16.8
Age		
18 to 30 years	25	20.0
31 to 40 years	58	46.4
41 to 60 years	42	33.6
Skin colour		
White	89	71.2
Black/brown	35	28.0
Yellow	1	0.8
Marital status		
Single	41	32.8
Married	71	56.8
Widower/divorced	13	10.4
Number of children		
None	65	52.0
One	30	24.0
Two or more	30	24.0
Educational level		
Primary school education	2	1.6
High school education	23	18.4
University	100	80.0
Body mass index (BMI)		
Underweight	1	0.8
Normal weight	50	40.0
Overweight	41	32.8
Obese	33	26.4
Smoke	14	11.2
Alcohol use more than 2 times/week	20	16.0
Physical activity during leisure time	69	55.2
Diagnosed disease	53	42.4
Medication use	83	66.4
Occupation		
Nurse	45	36.0
Nursing assistant/technician	28	22.4
Physiotherapist	26	20.8
Physician	8	6.4
Dentist	4	3.2
Other	14	11.2
Job seniority		
Less than 2 years	37	29.6
2–5 years	42	33.6
More than 5 years	46	36.8
Workplace		
Primary care	40	32.0
Hospital	61	48.8
Emergency care	12	9.6
Ambulatory care	9	7.3
Homecare	3	2.4
Weekly working hours		
Up to 24 h	6	4.8
30 h	30	24.0
36 h	21	16.8
40 h	60	48.0
>40 h	8	6.4
Other employment	39	31.2
Family income (US$)		
>1 to 3 × MMW	25	20.0
>3 to 6 × MMW	49	39.2
>6 to 9 × MMW	22	17.6
>9 × MMW	25	20.0
Not declared	4	3.2

MMW: monthly minimum wage = R 1045 ≅ USD 200.

**Table 2:** The prevalence of musculoskeletal symptoms among healthcare workers of the HEROES cohort at baseline. Data are expressed as % and 95% CI.

Body Region	12-Month Symptoms	12-Month Disability	12-Month Healthcare Assistance	7-Day Symptoms
Neck	64.0 (55–72)	20.0 (14–28)	18.4 (16–26)	31.2 (24–40)
Shoulders	62.4 (54–70)	13.6 (9–21)	13.6 (9–21)	26.4 (19–35)
Upper back	58.4 (50–67)	16.0 (11–23)	12.8 (8–20)	29.6 (22–38)
Elbow	9.6 (6–16)	3.2 (1–8)	6.4 (3–12)	5.6 (3–11)
Lower back	60.8 (52–69)	23.2 (17–31)	15.2 (10–23)	39.2 (31–48)
Wrist/hand	42.4 (34–51)	10.4 (6–17)	8.8 (5–15)	13.6 (9–21)
Hip/thigh	29.6 (22–38)	5.6 (3–11)	7.2 (4–13)	12.0 (7–19)
Knee	32.0 (24–41)	9.6 (6–16)	8.8 (5–15)	16.8 (11–24)
Ankle/foot	36.8 (29–46)	8.0 (4–14)	9.6 (6–16)	20.8 (15–29)

## Data Availability

The data that support the findings of this study are available upon request from the corresponding author, T.O.S.
